# Hearing Impairment and Risk of Depression in Older Adults in Health ABC

**DOI:** 10.1093/geroni/igaa057.1509

**Published:** 2020-12-16

**Authors:** Danielle Powell, Joshua Betz, Kristine Yaffe, Stephen Kritchevsky, Elsa Strotmeyer, Eleanor Simonsick, Frank Lin, Jennifer Deal

**Affiliations:** 1 Johns Hopkins University, Baltimore, Maryland, United States; 2 Johns Hopkins Bloomberg School of Public Health, Baltimore, Maryland, United States; 3 UCSF Weill Institute for Neurosciences, San Francisco, California, United States; 4 Wake Forest School of Medicine, Winston-Salem, North Carolina, United States; 5 University of Pittsburgh, Pittsburgh, Pennsylvania, United States; 6 National Institute on Aging, Bethesda, Maryland, United States; 7 Johns Hopkins University School of Medicine, Baltimore, Maryland, United States

## Abstract

Whether hearing impairment (HI) is associated with depressive symptoms remains disputed for older adults, in part due to varying definition employed, use of subjective hearing measures, or cross-sectional analysis. We studied 1936 men and women (mean age 74.1 years, 41.7% black race) enrolled in the prospective Health, Aging and Body Composition study Hearing thresholds at 500-4000 Hz were averaged to create a pure tone average (PTA) and HI was defined using clinical cutpoints in the better-hearing ear. Depression was measured using the Center for Epidemiologic Studies Depression Scale (CES-D) or the CES-D 10, a revised 10 question scale depending on visit. Linear mixed effects models with random intercepts and slopes were used to estimate difference in rates of change in depressive symptomatology by hearing status over nine years. Cox proportional hazard models were used to examine the association between HI and incident depression defined as change in CES-D score >=10 points. In models adjusted for demographic and clinical covariates, participants with HI demonstrated a higher baseline prevalence of depressive symptoms compared to those with normal hearing (20.7% vs. 8.4%).Rates of change did not differ by HI status. Participants with moderate or greater HI had an increased risk of 9-year incident depression (HR=1.28, 95% CI: 1.00-1.62) compared to participants with normal hearing. HI is associated with increased risk of incident depression and a greater overall prevalence of depression compared to normal hearing, underscoring the importance of further research on whether rehabilitative therapies can mitigate this association.

